# Enhancing the physicochemical qualities and consumer acceptability of rice noodles through yeast fermentation

**DOI:** 10.1002/fsn3.3803

**Published:** 2025-04-18

**Authors:** Shin‐Yong Yeoh, Nur 'Ain Syakirah Abdul Majid, Azhar Mat Easa

**Affiliations:** ^1^ Food Technology Division, School of Industrial Technology Universiti Sains Malaysia, USM Georgetown Malaysia

**Keywords:** fermented rice noodles, quality attributes, *Saccharomyces cerevisiae*, starch, yeast fermentation

## Abstract

Fermented rice noodles (FRN) are popular in many Asian countries due to their extraordinary flavor and mouthfeel. However, they havea short shelf life, inconsistent texture and sensory properties caused by uncontrolled microbial activity during fermentation. Furthermore, the lack of gluten contributes to their weak texture due to reduced cohesion and extensibility. The impact of yeast fermentation on the cooking, textural properties, microstructure, and consumer acceptability of cooked rice noodles (RN) was investigated. Yeast fermentation significantly enhanced the overall quality of cooked RN. FRN had a lower pH than conventional rice noodles (CRN), owing to the organic acids produced during fermentation. Notably, FRN subjected to a 3‐hour fermentation period (FRN3) achieved the highest cooking yield, shortest optimum cooking time, and lowest cooking loss among all RN samples. This achievement is linked to the creation of multiple irregularly sized pores within the starch network, resulting from carbon dioxide generation and starch hydrolysis by microbial metabolites during yeast fermentation. Yeast fermentation decreased lightness and increased yellowness in RN, altering their color attributes. FRN3 earned the highest score for overall acceptability in sensory evaluations due to its superior appearance, taste, and texture. These findings open new avenues for future research on using yeast starter culture to enhance rice products and contribute to developing improved RN products with desirable sensory and textural properties.

## Graphical abstract

Yeast fermentation significantly improved the quality of cooked rice noodles, with a 3‐hour fermentation period (FRN3) yielding the best results in cooking qualities and consumer acceptability. The fermentation process altered the pH, texture, and color of the noodles due to the production of organic acids, carbon dioxide, and microbial metabolites. These findings offer promising directions for using yeast cultures to develop enhanced rice products with improved sensory and textural properties.

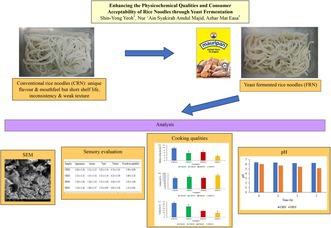

## 1 INTRODUCTION

The unicellular eukaryotic organism *Saccharomyces cerevisiae* (baker's yeast) has been widely exploited for its versatile qualities, including its high fermentative capability and ability to thrive in undesirable conditions of pH, temperature, osmolarity, and nutrient and ethanol availability. *S. cerevisiae* dominates fermentation because it can withstand high levels of ethanol that other microorganisms cannot tolerate (Pereira et al., 2021). Baker's yeast is a food‐grade yeast that is widely used in the baking, bread industry, and food and beverage fermentation worldwide (Lahue et al., 2020; Takalloo et al., 2020). It is an important source of yeast extract (Takalloo et al., 2020). It consists of 30%–33% dry materials, 40.6%–58.0% proteins, 35.0%–45.0% carbohydrates, 5.0%–7.5% minerals, 4.0%–6.0% lipids, and various vitamins (Heitmann et al., 2018). Yeast is promising for wide application as a probiotic in animal nutrition and health because of its potent antimicrobial and biosorption properties (Schiavone et al., 2015).

Globally, rice consumption has recently decreased because of changes in diet, which has spurred the development of a wide range of rice‐based foods created through industrial processing. Rice flour is widely used in baby food, puddings, noodles, and Asian dishes. It has also gained attention as a potential gluten‐free ingredient (Park et al., 2020). Fermented rice noodles (FRN) are well known, and their consumption is widespread in Asian countries (Yi, Zhu, Bao, et al., 2020). Low et al. (2020) reported that market research conducted by Grand View Research Inc. deduced that the rice noodles (RN) market in Europe and Asia Pacific in 2014 was USD 1.69 billion. This figure is expected to grow consistently to USD 3.6 billion by 2022. Despite their white color, FRN have a chewy and highly satisfactory mouthfeel (Li et al., 2019). RN texture and sensory characteristics are improved by both natural and lactic acid bacteria/yeast fermentation (Li et al., 2019; Yi et al., 2017). Some FRN undergo natural fermentation without the use of a starter culture (Yi et al., 2019).

The characteristics of *S. cerevisiae* make it ideal for traditional fermentation processes, as it produces extracellular enzymes and is non‐pathogenic and cost‐effective (Wang et al., 2022). The fermentation of starch by yeast strains yields simple sugars, ethanol, and carbon dioxide, which provide leavening effects in various foods (Li et al., 2019). Yeast has a significant influence on the flavor (by producing esters, aldehydes, and ketones), color (carbohydrates and amino acids), and shelf life (acids and glycerol) of baked goods (De Vuyst et al., 2016; Heitmann et al., 2018). These metabolites enhance the physical properties of dough, such as rheological qualities, softness, viscosity, and elasticity (Zhang et al., 2023).

The lack of gluten in RN contributes to the low elasticity, poor cohesion, resistance to stretching and mixing, and extensible properties of FRN when compared with wheat‐based noodles (Srikaeo et al., 2018). Wang et al. (2022) observed that the fermentation process initiated by four starter cultures that consisted of *Limosilactobacillus fermentum*, *Lactoplantibacillus plantarum*, and *S. cerevisiae* disrupted the organized crystal structures of starch while also breaking down proteins and lipids. As a result, the influence of the protein network on starch granules was diminished, ultimately contributing to the strengthening of the starch gel structure. Consequently, FRN demonstrated an increasing trend in their textural attributes. The traditional method of producing FRN depends on the microorganisms present in the rice material and the surrounding environment, resulting in a long fermentation period and posing a safety concern, resulting in product inconsistencies (Zhang et al., 2023). Furthermore, FRN contain a substantial amount of water (62.51%) and have a concise shelf life (2–3 days) (Low et al., 2020).

To address these issues, starter cultures comprising different microbes have replaced natural fermentation to provide RN with standardized attributes (Wang et al., 2023). Applying pure strains eliminates the drawbacks of natural fermentation, including uncontrolled strain constitution and undesired microbial contamination. Starters are microorganisms or their cultures used in fermentation to enhance the activity and consistency of fermentation strains (He et al., 2022). Several studies have shown that adding selected bacteria and yeast strains from fermentation cultures for fermentation enhances the safety and organoleptic properties of FRN and the effects of fermentation (Li et al., 2021; Wang et al., 2023). Wang et al. (2023) investigated the effect of *Limosilactobacillus fermentum*, *Lactoplantibacillus plantarum*, *Lactococcus lactis*, and *S. cerevisiae* on the edible quality, microbial community, and volatile component of fresh FRN. However, there has been no research investigating the effect of pure yeast (*S. cerevisiae*) fermentation from instant dry yeast (commercially available) on the textural, sensory quality, and microstructure of FRN. This study investigated the pH, microstructure, physical properties, cooking, and sensory quality of fresh RN fermented with yeast.

## 2 MATERIALS AND METHODS

### 2.1 Materials

Commercial rice flour (Alagappa Flour Mills Sdn Bhd, Penang, Malaysia) (~6% protein), Mauripan instant dry yeast, and salt were purchased from Lotuss Stores Malaysia Sdn Bhd (Lotus's Malaysia, Georgetown, Malaysia). Alagappa brand rice flour was selected because it is a commercial rice flour often used in Malaysia. Other chemicals used in this study (analytical grade) were purchased from Merck Sdn Bhd (Selangor, Malaysia).

### 2.2 Preparation of RN

**FIGURE 1:**
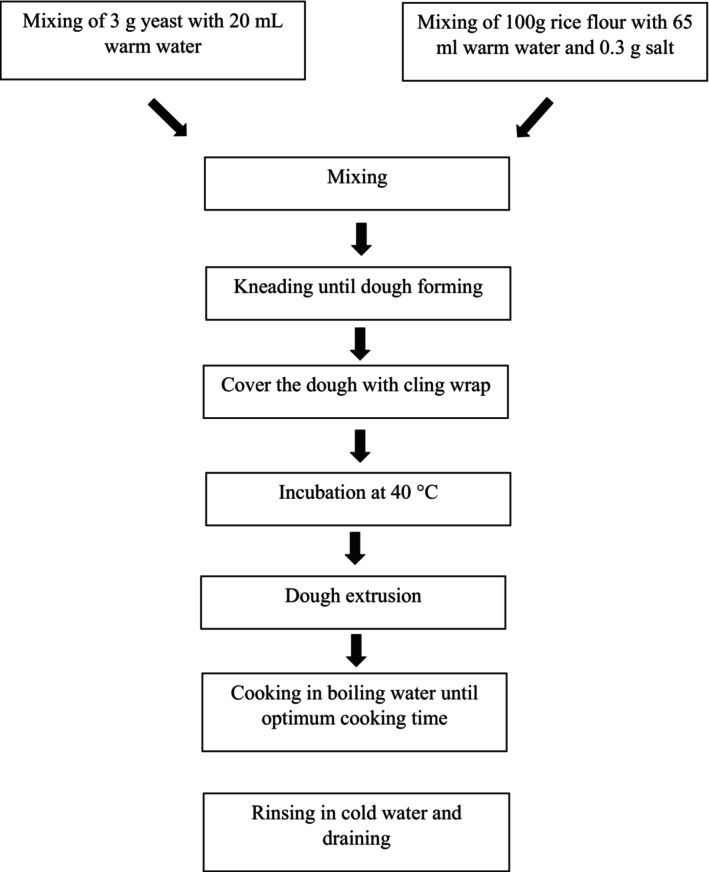
The flowchart for the preparation of CRN and FRN. CRN, conventional rice noodles; FRN, fermented rice noodles.

The RN were prepared according to Ojukwu et al. (2020) with modifications. Conventional RN (CRN) consisted of 100 g of rice flour, 85 mL of warm water, and 0.3 g of salt. The dough was prepared by mixing all the ingredients for 10 min using a food mixer (Spar Model 800‐C). To prepare FRN, 3 g of yeast was mixed with 20 mL of warm water before leaving the yeast solution for 10 min at the ambient temperature. Then, the yeast solution was added to a rice dough consisting of 100 g of rice flour and 65 mL of warm water. Next, the dough was kneaded until soft. The dough was covered with cling wrap and incubated at 40°C in an incubator (Carbolite, UK) at 2 fermentation times, which were 0 h (CRN0 and FRN0) and 3 h (CRN3 and FRN3) for all analysis except pH analysis. Next, the dough was extruded using a stainless steel pressure surface machine (Limai LM‐20). The RN were cooked in boiling water until their optimum cooking time was achieved. Then, the noodles were rinsed in cold water and drained. The noodles were placed on a plastic plate at room temperature for 15 min before analysis. The flowchart for the preparation of RN is presented in Figure 1.

### 2.3 Determination of pH

CRN and FRN were incubated in an incubator (Carbolite, UK) at four different times, which were 0, 1, 3, and 5 h. The pH values were determined using a Mettler‐Toledo Delta 320 pH meter calibrated with a pH 4.0 and 10.0 buffer solution (Yeoh et al., 2020). The samples (10 g) were homogenized for 5 min in 100 mL of deionized water and then filtered. pH analysis was conducted after 30 min of waiting.

### 2.4 Determination of noodles cooking quality

The cooking quality of the noodles was assessed by determining their optimal cooking time, cooking yield, and cooking loss (Yeoh et al., 2020). Each sample was analyzed in triplicate. First, ten strands of RN were boiled for 3 min in 300 mL of boiling water and then at 30s intervals or until the white center in RN disappeared. The optimum cooking time was determined when the white center of the noodles strands disappeared. The cooking yield was used to calculate the water holding capacity during cooking. The formula for cooking yield is:

$$\text{Cooking yield}\;\left(\%\right)=\frac{\text{Weight of noodles after cooking}}{\text{Weight of noodles before cooking}}\times 100$$

The weight of the solid loss after drying the cooking water from noodles to a stable weight in a 105°C oven was used to calculate the cooking loss. The formula for cooking loss is:

$$\text{Cooking loss}\;\left(\%\right)=\frac{A-B}{C}\times 100$$

where *A* is the weight of the beaker and dry cooked water sample (g), *B* denotes the weight of the beaker (g), and *C* corresponds to the weight of raw noodles (g).

### 2.5 Texture analysis

The texture of the RN was determined according to Ojukwu et al. (2020). The hardness and adhesiveness of cooked noodles samples were measured using a Texture Analyzer (Stable Micro Systems, Surrey, UK) with a load cell of 5 kg. A 36‐mm compression probe (P/36) was used, and the test settings were fixed at a compression strain of 75% of the original RN thickness. A load cell of 5 kg was used for calibration before starting the analysis. The distance of movement of the probe was 15 mm. The pasta firmness/stickiness rig was employed, where the cooked RN strands were arranged in parallel on a flat metal platform. The settings were as follows: test speed of 1 mm/s; trigger type, auto‐20 g; pre‐test: 2 mm/s; post‐test: 1 mm/s and interval of two compressions, 1.0 sec. Ten strands of noodles from each group were cooked, cooled, drained, and rested for 10 min at 25°C before analysis. Cooked noodles were independently measured by positioning one end in the bottom rig arm slot and turning the detached arm correctly to bind the end of the noodles. The arm was stiffened, and the exact steps were applied to grapple the other end of the noodles to the upper arm. The thickness of the noodles was calculated using a manual micrometer (Dial Thickness Gauge Mitutoyo MI 7305, Japan), whereas the width of the noodles was calculated using a meter ruler. Tensile strength was calculated as follows:

$$\sigma =\frac{F}{A}$$

where *σ* corresponds to the tensile strength (Pa), F represents the maximum load or peak force (N), and *A* is the cross‐sectional area of the noodles strand (m^2^). The cross‐sectional area represents the product of the thickness and width of the noodles. The elasticity modulus was determined according to the following equation:

$$\text{Elascitiy modulus}=\frac{Fl_{0}}{\mathrm{tA}}+\frac{1}{v}$$

where *F*/*t* is the initial slope (N/s) of the graph (Force vs. Time), *l*_0_ represents the original length of the noodles between the limit arms (0.015 m), *A* denotes the original cross‐sectional area of the noodles (m^2^), and *v* shows the rate of movement of the upper arm (0.003 m/s).

### 2.6 Color measurement

A colorimeter (Konica Minota, Model CM 3600d, USA) was used to measure the color of cooked RN samples (Lubowa et al., 2018). International Commission on Illumination (CIE) L* (lightness), a* (redness), and b* (yellowness) values were recorded at random locations on the noodles surface. Each sample was analyzed in triplicate.

### 2.7 Microstructure analysis

The microstructure of the samples of RN was identified using scanning electron microscopy (Quanta 650 FEG SEM, FEI Technologies Inc., US) (Yeoh et al., 2023). The noodles samples were freeze‐dried before analysis. The freeze‐dried noodles samples were then cut and placed on a sample holder with the cracked side facing out. The platinum coating on the attached noodles samples increased their conductivity, and the cross‐section of the noodles was examined with SEM at 300× magnification. The RN were examined at 5 kV. Ten random pore sizes were measured for each sample, and the average pore size was calculated.

### 2.8 Sensory evaluation

The sensory evaluation received ethical approval from the Universiti Sains Malaysia human ethics committee (code number: USM/JEPeM/22060388 4, Jawatankuasa Penyelidikan Manusia Universiti Sains Malaysia (JEPeM)). Informed written consent was obtained from all 44 panelists, who were Food Technology undergraduate and postgraduate students at Universiti Sains Malaysia. All panelists were well‐informed before the sensory evaluation. CRN0, CRN3, FRN0, and FRN3 were selected for this evaluation. Every sample was prepared in 5‐cm pieces before being cooking in boiling water at a ratio of 1:10 (one part noodles to 10 parts water) according to optimum cooking time. Every sample was kept in a covered container before being served with gravy in bowls in a 3–4 g portion, with a labeled 3‐digit random number and in random order. Each panelist was required to evaluate each of the RN samples for the five attributes (appearance, color, texture, taste, and overall acceptability) using a 7‐point hedonic scale with the following numerical values: 1 = dislike strongly, 2 = dislike moderately, 3 = dislike slightly, 4 = neither like nor dislike, 5 = like slightly, 6 = like moderately, 7 = like strongly (Lubowa et al., 2021). A cup of distilled water, tissue paper, and an empty cup were arranged for the panelists to rinse their mouths at the beginning of the evaluation and between the evaluations of each sample.

### 2.9 Statistical analysis

All analysis was conducted in triplicate. Texture analysis was carried out in ten replicates for all samples. Results were expressed as means and standard deviation and analyzed using SPSS Window, version 27.0 (*p* < .05) (SPSS Inc.). Comparisons of means were performed by one‐way analysis of variance (ANOVA), followed by Duncan's test, except for pH analysis. The data obtained from the pH analysis were analyzed using the independent samples *t*‐test through SPSS Window, version 27.0 (*p* < .05) (SPSS Inc., Chicago, IL, USA).

## 3 RESULTS AND DISCUSSION

### 3.1 Determination of pH value

**FIGURE 2:**
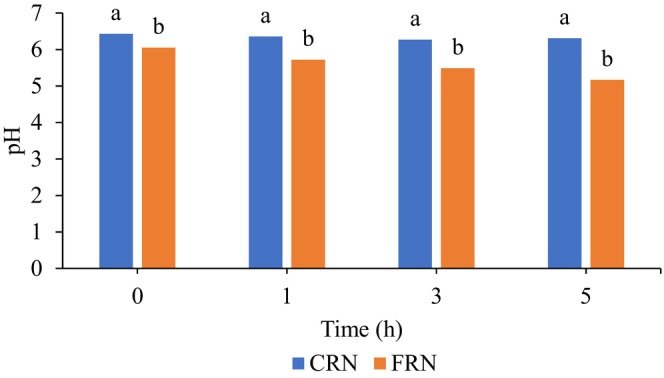
The pH change of CRN and FRN as a function of fermentation time at 25°C. CRN, conventional rice noodles; FRN, fermented rice noodles.

The pH values of CRN and FRN at 0 h were 6.43 and 6.05 (Figure 2), which are good for promoting fermentation. Wang et al. (2023) reported that the pH of fresh RN with *S. cerevisiae* at 0 h was ~6.5. The respiration of yeast cells consumed oxygen in the dough during mixing for a few minutes. Consequently, the anaerobic conditions created caused the proliferation of yeast cells to slow down and the fermentation reaction to occur. As more complex carbohydrates such as sucrose, maltose, and starch were broken down, glucose and fructose were produced and converted by yeast cells to carbon dioxide and ethanol (Parapouli et al., 2020). Liu et al. (2015) reported that fermentation in a more acidic environment (pH 2.75 and 2.50) inhibited yeast growth and reduced fermentation rates and the levels of acetic acid, glycerol, ethanol, and L‐succinic acid.

During the fermentation time of up to 5 h, the pH value of the FRN declined gradually. The pH of fresh RN with *S. cerevisiae* decreased to approximately 6.1 after 5 h of fermentation in Wang et al. (2023)'s study. Zhao et al. (2019) stated that succinic acid produced by yeasts and other organic acids during fermentation contributed to the decrease in pH. Furthermore, acidic substances accumulated as fermentation progressed. Jayaram et al. (2013) explained that the main contributor to the pH decline in wheat dough was succinic acid generated by fermenting yeast cells. The restricted carbon dioxide solubility in the liquid phase of the dough contributed to the limited formation of carbonic acid from the dissolved carbon dioxide. Acetic acid and lactic acid were detected in the fermented dough, but lactic acid originated from the flour and yeast preparations. Li et al. (2019) found that fermentation of the rice slurry with yeast resulted in a moderate pH decrease. Yeast fermentation improved the activity of microbial enzymes, food's nutritional value, and food digestibility because it maintained an acidic environment at a temperature of 22–25°C (Sharma et al., 2020). Niçin et al. (2022) discovered that pH 6 and 30°C were optimal for producing most bread flavor volatiles by *S. cerevisiae* to improve bread aroma characteristics. Mozaffary et al. (2019) reported that low pH was positively correlated with a low concentration of Ochratoxin A in the yeast‐fermented dough. Ochratoxin A (OTA) is a human carcinogenic and nephrotoxic mycotoxin that exhibits carcinogenic, immunotoxic, mutagenic, and neurotoxic properties in Group 2B (IARC, 2002).

In CRN, the pH value decreased gradually during the natural fermentation process from 0 to 3 h and remained constant thereafter. Wang et al. (2022) stated that environmental changes had a significant impact on natural fermentation, leading to variability and inconsistency in the microbial composition. They found out that the bacterial species in the natural fermentation group were most prevalent in the initial stages, suggesting that certain primitive bacteria could have originated from the rice or the processing environment. In their research, Yi, Zhu, Yang, et al. (2020) identified five bacterial species (*Lactobacillus*, *Burkholderia*, *Gluconacetobacter*, *Lactococcus*, and *Leuconostoc*) in RN fermentation liquid. *Lactobacillus* demonstrated its ability to produce acetic acid and lactic acid from carbohydrates (Gänzle, 2015; Wang et al., 2023). *Clostridium* was detected as the dominant bacteria in natural fermentation, according to Geng et al. (2019) and it could enhance the production of acids, esters, and ethers through the metabolism of complex carbohydrates and fermentation. In comparison to FRN, CRN demonstrated higher pH levels during fermentation for up to 5 h. This observation aligns with the findings of Wang et al. (2023), who also noted higher pH levels during a 6‐h natural fermentation of rice paste compared to other fermented samples comprising selected starter cultures. The fermentation time for RN was determined by monitoring pH values at four different time points: 0, 1, 3, and 5 h. Therefore, the fermentation times of 0 and 3 h were selected for further analysis, as the pH values of CRN decreased from 0 to 3 h.

### 3.2 Determination of cooking quality

**FIGURE 3:**
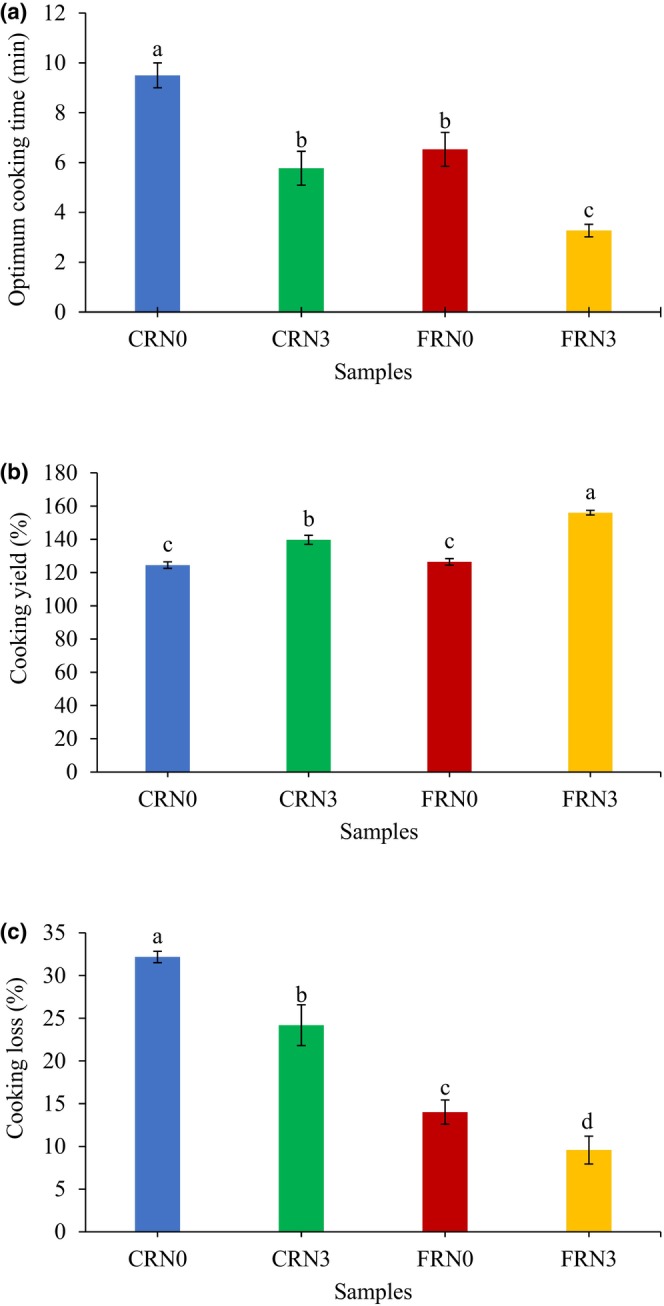
Cooking quality of cooked noodles. (a) Optimum cooking time; (b) cooking yield; and (c) cooking loss. Error bars indicate mean values ± standard deviations (*n* = 3) values. Different lowercase letters indicate significant differences (*p* < .05) in values between samples. CRN0, conventional rice noodles, underwent 0 h incubation; CRN3, conventional rice noodles, underwent 3 h incubation; FRN0, fermented rice noodles, underwent 0 h incubation; FRN3, fermented rice noodles, underwent 3 h incubation.

The cooking quality results are shown in Figure 3. Guo et al. (2023) defined the optimal cooking time as the amount of water absorbed by starch during cooking and the moisture content of the noodles. The optimum cooking time for CRN3 was significantly shorter than that for CRN0. The loss of moisture from the noodles surface during incubation could dehydrate the solid surface, thereby influencing the structure and optimal cooking time. FRN3 exhibited the shortest optimum cooking time among all samples. Lu et al. (2022) explained that the multiple pores were produced by the gas formed during fermentation, which aided the contact between water and starch during cooking, thus reducing the cooking time. They also reported that starch hydrolysis by microbial metabolites during the longer fermentation time of RN dough could contribute to water absorption, expansion, and starch gelatinization during noodles production. It responds to the demand for noodles that can be prepared quickly, as consumers opt for quick‐cooking RN (Srikaeo et al., 2018b). High‐quality RN exhibit short cooking times (Lu et al., 2022). In addition, the gas production rate increased over time. Thus, more holes were formed, and the absorption rate of water into the RN was accelerated.

Water is a critical factor affecting the quality and stability of RN. It is involved in various chemical, enzymatic, and physical degradation reactions and is necessary for the growth of spoilage organisms (Xue et al., 2021). The thickness of RN were 2.52–2.56 mm. The cooking yield of RN is shown in Figure 3b. FRN3 scored the highest cooking yield among all samples. The result aligns with the findings of Wang et al. (2023), in which RN with *S. cerevisiae* demonstrated the greatest water absorption. This might be attributed to the *S. cerevisiae* group noodles, which had the lowest protein content (with over 90% being hydrophobic protein from rice), allowing them to retain more water in their internal structure. They suggested that the interaction between water and other elements (total starch and protein) in FRN influenced the water absorption. FRN possessed a porous structure, facilitating water molecules' diffusion into the matrix during cooking. Additionally, starch present in the matrix absorbed more water (Lu et al., 2022). In addition to the severity, the orderly structures of rice, such as starch crystallites, were damaged by the fermentation process, which promoted the uptake of water molecules by the rice matrices. As a result, rice structures became less resistant to hydrothermal effects during steaming, resulting in a higher swelling level (Li et al., 2019). Furthermore, microbial metabolites hydrolyzed the starch during fermentation, which could facilitate water absorption, expansion, and gelatinization during cooking (Lu et al., 2022). All of these occurrences could have contributed to the short cooking time (Figure 2a). Sun et al. (2022) discovered that amylopectin short‐chain content increased remarkably while the long chains reduced due to the hydrolysis effect of enzymes in the starch fermented by yeast. Short amylopectin chains were positively correlated with a higher starch swelling power (Li & Zhu, 2017). Interestingly, CRN3 had a higher cooking yield than that of CRN0. The incubation time probably eliminated a certain amount of moisture from the noodles surface, affecting the external structure of the noodles strand and increasing water penetration into the noodles core. Furthermore, the structural compactness and porosity of the starch noodles might also play a role (Yang et al., 2021).

The cooking loss of the noodles is shown in Figure 3c. There was a significant difference (*p* < .05) in cooking loss among all the samples. Lubowa et al. (2021) explained that the solubilization of loosely bound gelatinized starch on noodles surfaces determined the cooking loss. However, it could vary depending on the degree of starch gelatinization and the strength of the starch network around the gelatinized starch. The cooking losses of both FRN were significantly lower (*p* < .05) than those of CRN. The results are consistent with the findings of Wang et al. (2023), where naturally fermented noodles exhibited a higher cooking loss. Lu et al. (2022) stated that microorganisms or their metabolites hydrolyzed starches and promoted amylose leaching during cooking after fermentation. This event could accelerate the interaction between starch and the enzyme, thereby influencing in vitro starch digestion. FRN3 had the lowest cooking loss, which could be attributed to the relatively short optimum cooking time. This result agrees with the report by Lu et al. (2022), where yeast‐fermented hollow noodles exhibited the lowest cooking loss among all samples. The cooking loss of starch noodles should not exceed 10% (Lubowa et al., 2018). In our study, the cooking loss of FRN3 was less than 10%, which is acceptable. However, the cooking losses of both CRN were greater than 20% due to the absence of gluten networks in the structure, and the encapsulation of starch polymers in the matrix was less effective. These results suggest that the addition of a single strain can enhance the cooking quality of RN, which is consistent with Wang et al.'s (2023) work.

### 3.3 Texture analysis

**FIGURE 4:**
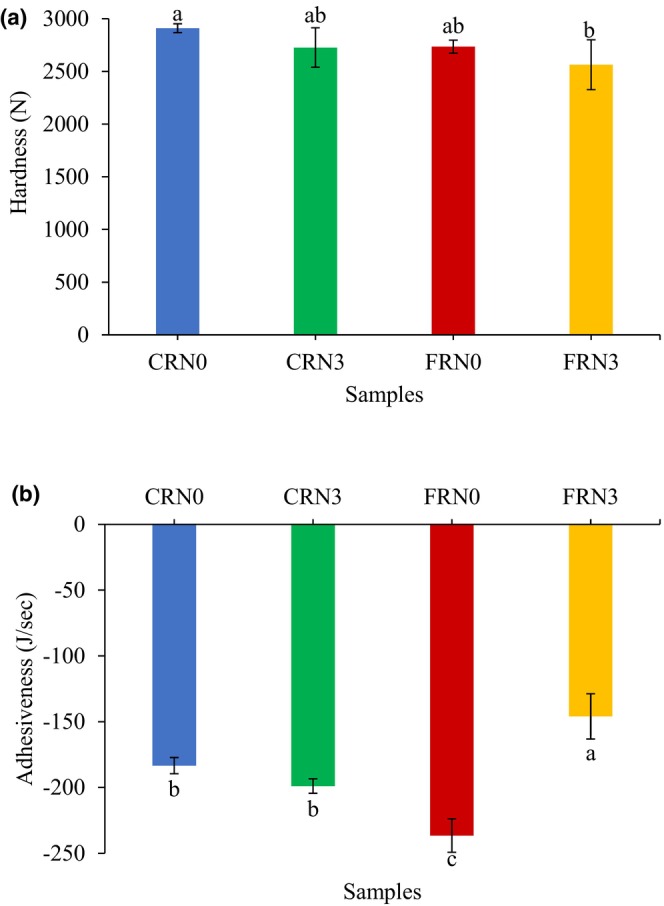
Textural parameters of cooked noodles at optimum cooking time. (a) Hardness and (b) adhesiveness. Error bars indicate mean values ± standard deviations (*n* = 10) values. Different lowercase letters indicate significant differences (*p* < .05) in values between samples. CRN0, conventional rice noodles, underwent 0 h incubation; CRN3, conventional rice noodles, underwent 3 h incubation; FRN0, fermented rice noodles, underwent 0 h incubation; FRN3, fermented rice noodles, underwent 3 h incubation.

The textural properties (hardness and adhesiveness) of CRN and FRN are shown in Figure 4. Hardness was in the order of FRN3 ≤ FRN0 < CRN3 ≤ CRN0. FRN3 showed the lowest hardness among all the samples, which could probably be attributed to the porous structures formed by carbon dioxide during yeast fermentation and the hydrolysis of starch by microbial metabolites, influencing the textural properties of the noodles. This phenomenon affected the water update of the noodles matrix during cooking and increased the cooking yield (Figure 3b). Xiong et al. (2021) discovered that yeast‐fermented hollow noodles acquired lower hardness values but higher adhesiveness values than non‐fermented noodles in their study. He et al. (2022) reported that 6–8 h fermentation times induced lower hardness and sensory quality during rice cake fermentation. CRN3 possessed a lower hardness score than CRN0, possibly because of the formation of a weaker gel structure after incubation. Some moisture was removed from the noodles surface during incubation, probably leading to dehydration of the solid surface and affecting the noodles texture (Ismail et al., 2021). The minimal changes in hardness data could possibly be attributed to the absence of gluten in rice flour, which affected the ability to form a consistent viscoelastic dough, thereby contributing to the unsatisfactory texture of RN (Ojukwu et al., 2021). The changes in structure and texture could be related to the changes in composition in starch, protein, lipid, and pH. According to Wang et al. (2023), the protein content in the RN containing *S. cerevisiae* decreased from ~7.3% to ~5.35%, whereas the lipid content dropped significantly from ~1.28% to ~0.68%, likely because the microorganisms converted the lipids into fatty acids. Additionally, the total starch content decreased from ~78% to 66% in the RN with *S. cerevisiae*. The fermentation process facilitated the transformation of starch, protein, and lipids into different compounds, which could lead to variations in flavor.

Noodles adhesiveness is a measure of the energy required to remove the test probe from adhered noodles and is an indicator of food smoothness (An et al., 2022). The adhesiveness of the RN is shown in Figure 4b. FRN3 demonstrated the highest adhesiveness, suggesting low smoothness, as adhesiveness was adversely associated with smoothness (An et al., 2021). Additionally, a positive correlation existed between the stickiness and adhesiveness of RN, and high stickiness was unfavorable (Kasunmala et al., 2020). A study by Yue et al. (2019) showed that yeast generated carbon dioxide and ethanol during proofing, which increased the stickiness of dough due to the longer fermentation time. Furthermore, they explained that the water content increased during fermentation, resulting in a higher cooking yield (Figure 3b) and adhesiveness values in our study. Zou et al. (2015) found that starch granules on the noodles surface had a higher rate of expansion and gelatinization than starches in the core area during cooking. Thus, the smoothness of the noodles was determined by the swelling of starch granules near their surface. An et al. (2021) proposed that high‐quality noodles should be smooth and low adhesive. They discovered that cooked noodles exhibited reduced adhesiveness after washing, suggesting that the smoothness of the noodles increased after washing because of the removal of material from the surface of the noodles. They concluded that the long amylopectin chain content increased noodles smoothness and adhesiveness.

Environmental factors and a varied and unpredictable bacterial population influence the natural fermentation. Consequently, the texture, sensory attributes, and food safety risks associated with RN may display inconsistencies, leading to significant differences in product characteristics across various regions (Geng et al., 2019; Wang et al., 2023).

### 3.4 Color analysis

**FIGURE 5:**
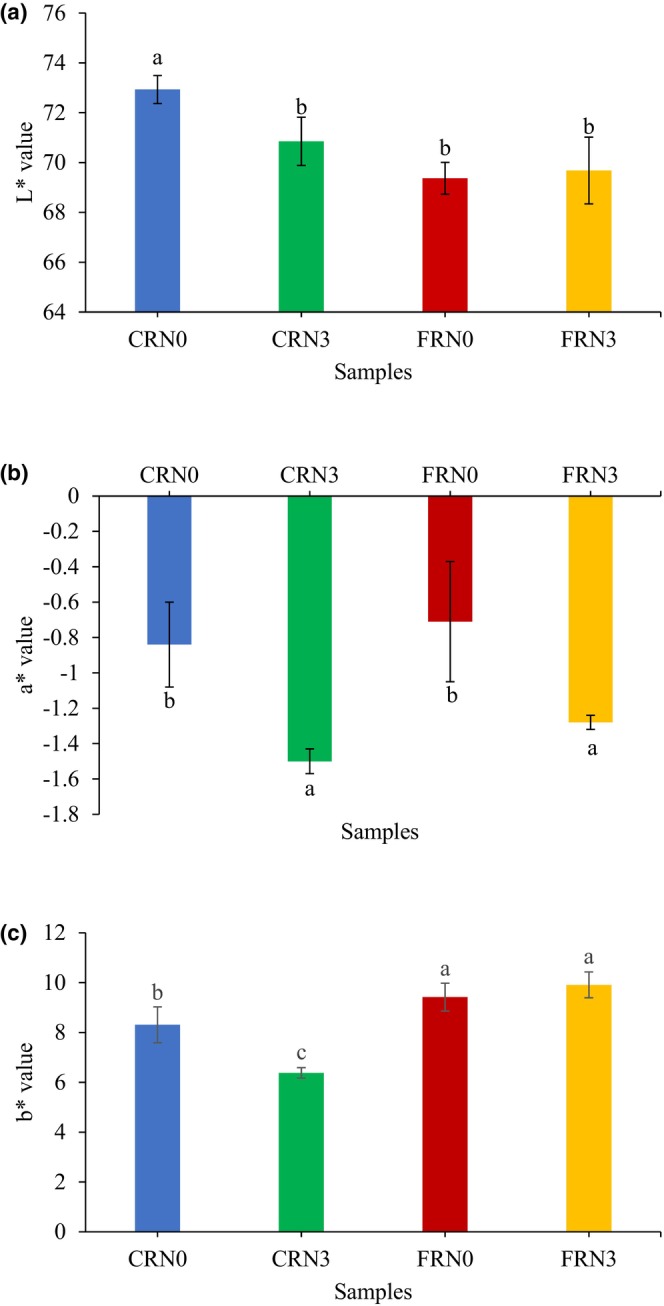
Color values of cooked noodles samples. (a) L* (lightness), (b) a* (redness), and (c) b* (yellowness). Error bars indicate mean values ± standard deviation (*n* = 3) values. Different lowercase letters indicate significant differences (*p* < .05) in values between samples. CRN0, conventional rice noodles, underwent 0 h incubation; CRN3, conventional rice noodles, underwent 3 h incubation; FRN0, fermented rice noodles, underwent 0 h incubation; FRN3, fermented rice noodles, underwent 3 h incubation.

**FIGURE 6:**
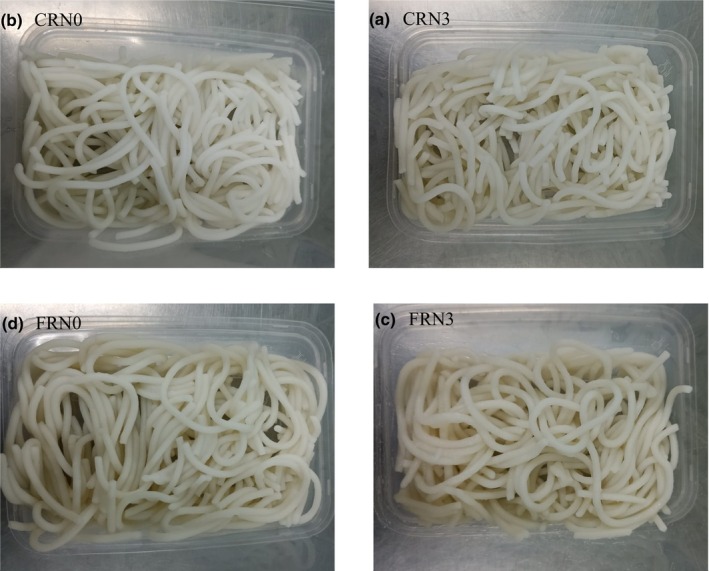
Color appearance of RN. (a) CRN0, (b) CRN3, (c) FRN0, and (d) FRN3. CRN0, conventional rice noodles, underwent 0 h incubation; CRN3, conventional rice noodles, underwent 3 h incubation; FRN0, fermented rice noodles, underwent 0 h incubation; FRN3, fermented rice noodles, underwent 3 h incubation.

RN color is a critical visual quality criterion, and it also determines the product's overall acceptability (Ojukwu et al., 2020). Consumers preferred RN with higher L* and lower b* values because they had lighter and glossier appearances (Xiao et al., 2022). Figure 5 illustrates the results of the color analysis of the RN. There was a significant difference (*p* < .05) in the L* (lightness), a* (redness), and b* (yellowness) values between the samples. The color of RN was a result of starch‐protein interactions, and RN should ideally appear white (Ojukwu et al., 2022). The L* value exhibited by CRN0 was close to that reported by Ojukwu et al. (2020), with fresh RN being the lightest among all samples. CRN3 possessed a significantly lower L* value than CRN0. A possible explanation could be the higher water update (Figure 3b) affecting texture and lightness. In FRN0 and FRN3, the lower lightness values could be attributed to the addition of yeast to the formulation, which decreased the lightness of RN. CRN3 and FRN3 yielded decreased a* values, possibly due to the leaching of the soluble colored compounds from the Maillard reaction and enzymatic browning during cooking, as both noodles possessed higher cooking yields than CRN0 and FRN0 (Figure 3b). The decrease in the b* value of CRN3 suggested that the yellow bran pigments in the rice flour leached into the cooking water with higher water uptake by CRN3 (Lamberts et al., 2006). The highest b* values in FRN0 and FRN3 were expected because yeast addition imparted yellowness to RN. No significant difference was observed in the b* values between FRN0 and FRN3. The color appearances of CRN0, CRN3, FRN0, and FRN3 are shown in Figure 6.

### 3.5 Microstructure analysis

**FIGURE 7:**
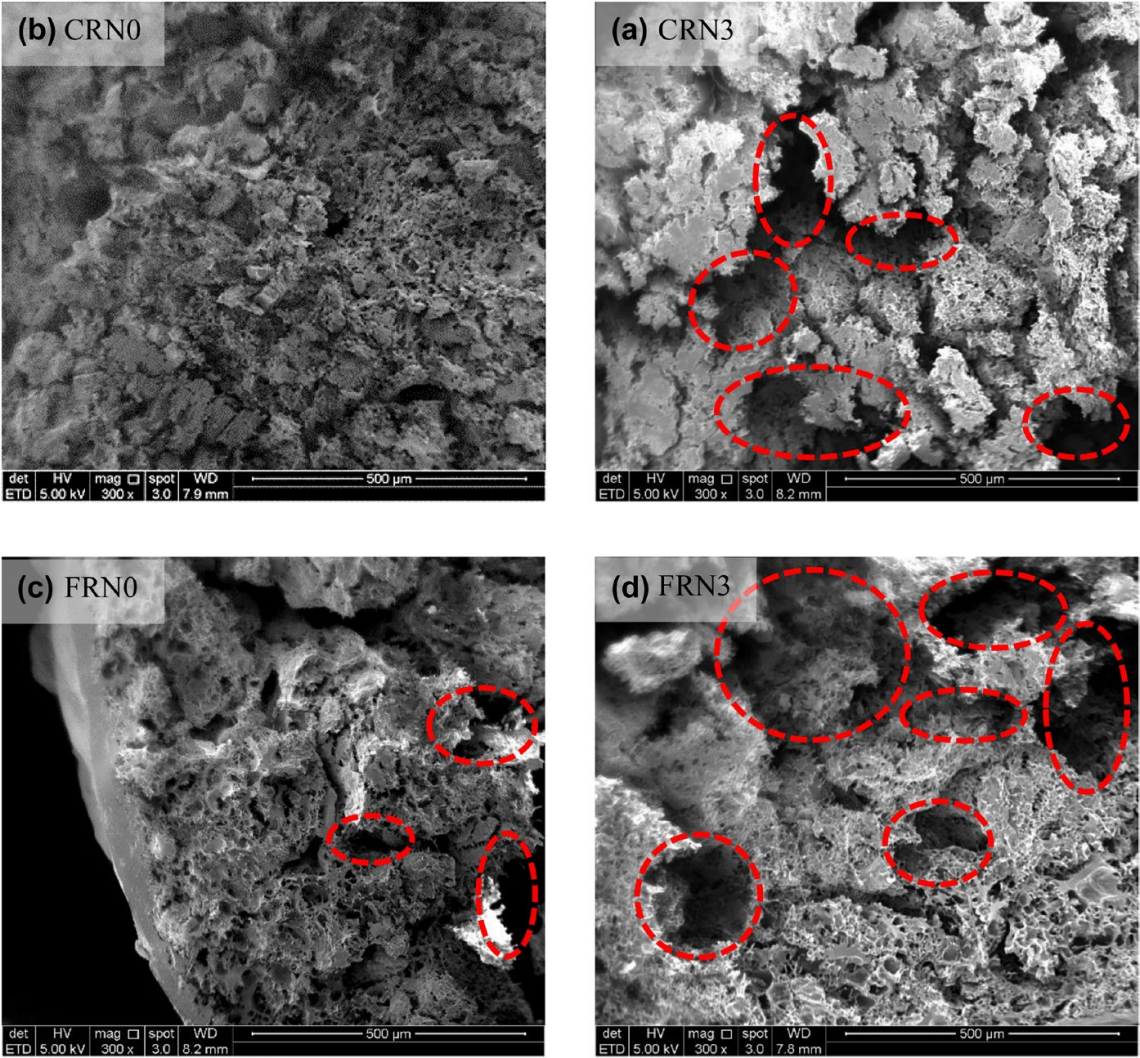
SEM images of RN samples at 300× magnification. Scale bar = 500 μm. (a) CRN0, (b) CRN3, (c) FRN0, and (d) FRN3. The circles in the images represent cracks in the noodles. CRN0, conventional rice noodles, underwent 0 h incubation; CRN3, conventional rice noodles, underwent 3 h incubation; FRN0, fermented rice noodles, underwent 0 h incubation; FRN3, fermented rice noodles, underwent 3 h incubation.

Figure 7 shows the porous structural variations at the cross‐sections of the four noodles. Starch formed micellar networks when heated due to the interaction between amylose and amylopectin, which governed the swelling process (Srikaeo et al., 2018b). CRN0 (Figure 7a) possessed a denser and more compact structure with smaller pores (12.31 ± 2.92 μm), which contributed to its highest optimum cooking time (Figure 3a) and hardness score (Figure 4a). In the study by Tu et al. (2021), native starch granules had a non‐uniform shape but were consistent in size and smooth surface. Yang et al. (2021) discovered a honeycombed network with firm interactions in cooked wet‐starch noodles. They also summarized that gelatinized starch morphology, particularly the size and number of internal pores, could comprehensively demonstrate the molecular interactions. Moreover, the honeycomb network influenced the hardness of the starch gel. Some pores (22.96 ± 3.51 μm) were observed in CRN3, and the molecules in the starch network exhibited weak interactions (Figure 7b). The microstructure of CRN3 is most likely responsible for the short optimum cooking time (Figure 3a), high cooking yield (Figure 3b), and low hardness value (Figure 4a) in our study. Similar observations were reported by Yang et al. (2021), who detected abundant pores with irregular sizes and poor networks in cooked dried starch noodles.

FRN0 (Figure 7c) exhibited more pores (31.51 ± 3.99 μm) and cracks than CRN0. These holes were formed by air bubbles of carbon dioxide during the mixing of the dough and yeast (Yue et al., 2019). Enzymes first attacked the starch granules to form pores, and the external part of the starch was subsequently damaged (Sun et al., 2022), likely causing the cracks. FRN3 (Figure 7d) was less compact and contained larger pores (58.9 ± 15.5 μm) and cracks than FRN0 because longer fermentation times led to excessive gas production and more damage to the external starch layer, resulting in cracks (Yue et al., 2019). There were a greater number of bigger holes observed in FRN3 when compared to CRN3 (Figure 7b). The increase in carbon dioxide in the gas cell probably disrupted the cell walls, allowing the gas to escape through the starch structure in FRN3. Furthermore, the ability of the dough to hold gas decreased over time (Yue et al., 2019). Tu et al. (2021) reported that the surface of the starch granules underwent significant disruption and erosion because of the fermentation process. They explained that the changes observed in starch granules could be due to microorganisms' hydrolysis of starch granules. Starch granules on the rice surface were destroyed by yeast, as reported by Li et al. (2019). The starch granules and porous structure in FRN3 enhanced water permeation into the noodles core, resulting in the highest cooking yield (Figure 3b) and a decreased hardness value (Figure 4a).

### 3.6 Sensory evaluation

The five sensory qualities of cooked RN are appearance, aroma, taste, texture, and overall acceptability. No attributes were significantly different (*p* > .05) between the samples (Table 1). Noodles products are usually purchased by Asian consumers from convenience stores or local manufacturers according to their initial evaluation of their quality based on their visual appearances, such as color, lightness, and absence of undesirable particles (Ahmed et al., 2015). Li et al. (2021) emphasized that RN should appear whiter and more transparent. Moreover, any RN that was dark or gray was unacceptable (Xue et al., 2021). FRN had slightly higher appearance scores than CRN, suggesting that panelists preferred the appearance of FRN. Yeast addition produced yellowness in RN, which improved the color, as evidenced by the higher b* values in the color analysis (Figure 5b).

**TABLE 1:** Sensory evaluation of cooked noodles.

Samples	Appearance	Aroma	Taste	Texture	Overall acceptability
CRN0	4.94 ± 1.18	5.12 ± 1.15	5.03 ± 1.14	4.74 ± 1.14	5.09 ± 0.99
CRN3	5.23 ± 1.23	5.15 ± 1.31	5.15 ± 1.54	4.23 ± 1.39	5.06 ± 1.20
FRN0	5.26 ± 1.16	5.18 ± 1.24	5.12 ± 1.04	4.44 ± 1.35	5.03 ± 1.06
FRN3	5.50 ± 1.16	5.06 ± 1.37	5.13 ± 0.95	4.85 ± 1.16	5.21 ± 0.91

*Note*: Results display mean values ± standard deviation (*n* = 44) values. No significance was observed between samples. CRN0, conventional rice noodles, underwent 0 h incubation; CRN3, conventional rice noodles, underwent 3 h incubation; FRN0, fermented rice noodles, underwent 0 h incubation; FRN3, fermented rice noodles, underwent 3 h incubation.

Rice variety and other ingredients could affect the aroma and taste of foods (Lubowa et al., 2021). The similarity in aroma and taste among all the samples was possibly due to the same rice variety used in our study. The aroma of cooked noodles exerted little influence on consumer acceptance of the product because it was not a major quality attribute compared with other aspects of the product (Ahmed et al., 2015). The panelists noted that the yeast aroma was more pronounced in FRN3, resulting in a lower sensory rating for aroma quality. Wang et al. (2022) investigated alcohols, aldehydes, acetoin, esters, and some flavor compounds generated in the starter culture. They summarized that the sour taste of RN was attributed to the ability of *S. cerevisiae* to convert aldehydes to acids. However, the sour taste in FRN3 might not have been recognized by the panelists because the taste score of FRN3 was similar to that of other RN. The flavor of CRN3 was most probably imparted by the natural fermentation, which comprised complex microbial and enzymatic processes. During natural fermentation, lactic acid bacteria metabolized carbohydrates to generate lactic acid and acetic acid. These compounds inhibited the growth of undesirable bacteria and contributed to the flavor of the fermented products. Furthermore, *Clostridium* could break down complex carbohydrates and fermentation byproducts, resulting in the production of both acids and esters (Geng et al., 2019; Lu et al., 2003). The order of texture was FRN3 > CRN0 > FRN0 > CRN3. Although FRN3 possessed high adhesiveness value (Figure 3b), the panelists preferred FRN3 over other RN; most likely, they preferred softer RN because of its low hardness (Figure 3a). In Lu et al.'s (2003) sensory evaluation, FRN exhibited a pleasant chewy mouthfeel after fermentation.

In terms of overall acceptability, FRN3 received the highest score from panelists. This was expected because FRN3 possessed a superior appearance, taste, and texture. Li et al. (2019) reported that the *Candida santamariae‐*treated noodles exhibited excellent sensory characteristics (e.g., color, aroma, taste, and mouthfeel) and achieved a higher total sensory score than untreated noodles in their study.

## 4 CONCLUSION

Yeast fermentation improved the cooking, textural, and sensory qualities of RN. FRN had a lower pH than CRN because of the production of organic acids during fermentation. FRN3 showed the highest cooking yield, shortest optimum cooking time, and lowest cooking loss among all the RN, as evidenced by the multiple pores with irregular sizes within the starch network. Moreover, the porous structures formed by carbon dioxide during yeast fermentation and the hydrolysis of starch by microbial metabolites affected the textural characteristics of RN and contributed to the low hardness of FRN3. In terms of color, yeast fermentation reduced the lightness and increased the yellowness of RN. The overall acceptability of FRN3 in the sensory evaluation was the highest because of its excellent color, taste, and texture. This study establishes a foundation for future research on yeast starter culture to improve rice products.

## AUTHOR CONTRIBUTIONS

**Shin‐Yong Yeoh:** Data curation (equal); formal analysis (equal); investigation (equal); methodology (equal); project administration (equal); software (lead); validation (lead); visualization (lead); writing – original draft (lead). **Nur 'Ain Syakirah Abdul Majid:** Data curation (equal); formal analysis (equal); investigation (equal); methodology (equal); visualization (supporting); writing – original draft (supporting). **Azhar Mat Easa:** Conceptualization (lead); funding acquisition (lead); project administration (equal); resources (lead); supervision (lead); validation (supporting); visualization (supporting); writing – review and editing (lead).

## CONFLICT OF INTEREST STATEMENT

The authors declare that they have no conflicts of interest.

## ETHICS STATEMENT

The sensory evaluation received ethical approval from the University human ethics committee (code number: USM/JEPeM/17050264, Jawatankuasa Penyelidikan Manusia Universiti Sains Malaysia (JEPeM)). Informed written consent was obtained from all human participants.

## Data Availability

Data sharing is not applicable to the main text. The data supporting the findings of this study are available on request from the corresponding authors.
